# Blood Examinations1Read before the section on pathology, Buffalo Academy of Medicine, September 18, 1900.

**Published:** 1900-12

**Authors:** H. G. Matzinger

**Affiliations:** Buffalo, N. Y., Professor of clinical pathology in University of Buffalo


### BLOOD EXAMINATIONS.[1]

By H. G. MATZINGER, M. D., Buffalo, N. Y.,
Professor of clinical pathology in University of Buffalo.

THE importance of the study of the blood can hardly be overestimated, when we consider that at one time or other, it carries all that is required as nutriment for all tissues of the body, as well as all the end products of constructive and destructive cell metabolism. Nothing in the whole range of biology offers such a promise of reward to the student nor confronts him with such an array of difficulties to overcome. There is no other field in the science of biology in which more careful and earnest work is being done. Out of all the mass of work of the past years the clinician has, however, gathered but little that he feels may not be contradicted tomorrow or that he

1. Read before the section on pathology, Buffalo Academy of Medicine, September 18, 1900.

can verify and put to every day use. This is what the general practitioner persuades himself to believe, and coupled with the difficulties of technique, has made him so out of patience with blood examinations, that he fails to avail himself of the help it can give him. It seems to me that he will take an entirely different view of the matter if he acquaints himself with the sources of error and the shortcomings of instruments, so doing he can get results and interpret them better, and will soon come to consider a blood examination as important as an urinalysis. If I can give ever so little help in that direction the purpose of this paper is attained.

Large expectations were entertained as to the clinical value of counts and microscopical examination of the cellular elements, but they failed to throw much light on conditions considered most promising, while some valuable help has been obtained where least expected. No one would have suspected that it would be of any help, *e.g*,, in the diagnosis of central pneumonia, deep-seated suppuration and trichinosis, or in the prognosis of relapsing fever, or of pneumonia. Yet with the confusion still existing in the results of trustworthy observers in color analysis of the white corpuscle, it offers a ready-made absolute diagnosis only in some five or six diseases. In the rest it is only a valuable part of the symptom complex presented by a disease, and ranks no higher in value in making a diagnosis than any other typical symptom. On the other hand, it is safe to insist that there are few diseased conditions, certainly none associated with fever, on which the examination of the blood may not throw some light.

The conditions in which a blood count and hemaglobin estimation are by all odds of greatest immediate and certain use in saving life are just the ones in which they are least employed. This is in emergency work in surgery. Any one who is moderately expert in the technique can obtain definite information within ten or fifteen minutes and without a microscope, information that will not only do away with the embarrassing doubt as to whether an operation caused death, or the condition for which operation was risked, but will save life.

Such cases are, *e.g.*,, the common ones in accident hospitals, in which it seems impossible to determine whether the extreme condition of the patient is due to shock, concussion or compression, or whether it is due to hemorrhage. In the one case transfusion will save, and operation will kill, in the other the reverse results will obtain.

Internal concealed hemorrhages, such as may occur in tubal rupture, in extrauterine pregnancy, can be made out with certainty, easily excluding peritonitis, appendicitis, obstruction or strangulated hernia. Any other concealed bleeding from organs can be detected as well and the location made out with the aid of the physical signs.

A very safe rule to follow is never to operate when hemaglobin is as low as 30 per cent. A comparatively short time is required to regenerate the blood, if phthisis, malignant disease, typhoid, etc., are not complicating the case. Other things being equal, less than 1 per cent. of ,the blood mass is made up in two to five days, from 1 to 3 per cent. in five to fourteen days and from 3 to 4 per cent., which would mean very severe hemorrhage, is made up in from two weeks to one month.

The life of the red cells is very brief, even under normal conditions. There is, of course, no way of determining it accurately, but physiologists are agreed that it is no longer than three weeks, if as long, and that it may vary much in different individuals. The mass of pigment continuously excreted in urine and feces is hemoglobin in oxidized and hydrated form. In all probability some of the iron of the destroyed red cell is used over and over again in the manufacture of new corpuscles, but the bulk is excreted so that there must be a constant wholesale destruction and consequent generation of red cells going on normally all the time. This, with the fact that under ordinary conditions loss of blood is quickly made up without symptoms of trouble, is proof positive that the red cell is very short lived. Of the life history of white cells much less is known. It seems quite likely that the different forms may be different stages in its life history, and yet this cannot be proven; indeed, it cannot be denied that some forms at least, may multiply as all other cells do by karyokinesis, producing the same as well as differnt types. There is no charnel house for them like the spleen for reds. As a matter of fact it is not known what becomes of them. Some subtle influence, of which nothing is known now, is constantly at work keeping the number of both at a certain point, provided the physical conditions are the same and are normal. The same is true of the bulk of blood, notwithstanding the fact that periodically in the day large quantities of fluid of one kind or other are absorbed into the blood vessels. When we consider that the lymphatic system of vessels carrying twice the bulk of the blood in lymph, is directly connected at both ends, so to say, with the general circulation and that the flow of lymph is not uniform, it becomes still more astonishing that the mass of blood and the proportion of corpuscles, especially reds, remains so nearly stationary.

The condition known as plethora, cannot exist for any length of time, nor can the opposite condition. Both are associated with very grave symptoms when produced experimentally or by disease, as acute failure of compensation in heart disease or vaso-motor paralysis. A temporary serous plethora or dilution can be brought about by transfusion of fluid in large amounts or its introduction by mouth or rectum, but is very promptly changed by diuresis and diaphoresis. A temporary cellular plethora or polycythemia is sometimes seen in rapid blood regeneration during recovery from severe anemia, and for a short time after transfusion of blood. Temporary concentration has been observed when the diffusible elements of the blood, chiefly water, are rapidly carried away by such causes, as profuse sweating or purgation, acute diarrhea and deprivation of liquids.

Similar but fleeting effects can be produced by vessel constricting influences, pain, cold, emotional, or vaso-motor disturbances, supra-renal extract, and the like, but they last at most but a short time. Constant normal changes in blood concentration and dilution, even hemaglobin percentage can, however, apparently be determined to some degree by physical conditions, such as altitude, atmospheric pressure and conditions of humidity. They obtain only as long as these unusual conditions of environment continue. Hot and cold baths also materially affect the proportions of the blood. The former giving a higher count, the latter a lower in reds with increase of whites, and the change may continue half an hour or longer.

The more profound physiological changes are easily dealt with, but the lesser ones are often overlooked and when considered their value is difficult to gauge in the correction of observations made, and when further complicated, perhaps augmented, by the effect of disease processes, may make the single determination of corpuscles and hemoglobin of doubtful diagnostic value.

In most individuals there is a normal variation in red cells and hemoglobin during the twenty-four hours. For hemaglobin there is an average rise of 7.2 per cent. during the night and a fall of 7.4 per cent. during the day. This occurs in about 88 per cent. of normal individuals. Sometimes the rise and fall amount to 20 per cent.; sometimes there is no change. At other times in the same individuals the conditions are reversed, but then the variations amount to less than 2.5 per cent. The percentage of red cells follows that of hemaglobin, but is not so marked, and here the day fall is more frequent and marked than the night rise, and the times when no change occurs at all is greater in proportion. In all probability these variations depend more on the changes in the plasma than on an actual change in number of corpuscles, and if that be so, the changing proportion of hemaglobin in the chromocytes or red cells, which is the greatest change of all, is the thing to be accounted for and studied. We should consider, then, that the worth and number of the chromocytes is an unstable one, even in normal individuals, and is at times found to be above or below the mean.

There are subsidiary changes of rise and fall as well, due to work, digestion and absorption of food, especially marked in the last third of the waking hours. The condition before breakfast and before lunch are almost equal, so that this time of day is the ideal one for making observations.

During digestion the average worth of the chromocytes remains unchanged, but for two or three hours after there is a distinct fall in both cells and hemogobin in 70 per cent. of cases, a rise in 23 per cent. and no change in 7 per cent. (100 meals—B. L. and D.—of same weight and ingredients). In 47 per cent. there is a slight rise in worth at the beginning immediately after the meal, and also at the very end of the fall. This effect of digestion is probably mostly due to the fluid transfer—not of fluids ingested, however, and the hemolytic and hemopoietic function of the accessory organs of primary digestion. Perhaps the preliminary rise may be due to formation of digestive fluids. But this is quickly compensated for. The general fall is associated with fall in specific gravity, which is promptly corrected by renal elimination. Strange to say, the amount of fluid food taken has less effect on this fall of blood decimal than the kind of solid food taken.

The blood taken from different parts of the body surface does not always give the same results—indeed, often there is quite a difference in the percentage of cells and hemoglobin. Certainly it is not the same in all individuals. The blood of the toe is often not so rich as that of the finger, doubtless due to the fact that gravity keeps the lymph longer in dependent portions. Massage of the toe will bring the percentage up (see chart). Gravity has much to do with the composition of the blood. Experiments in posture changes prove this. Raising of the arm into horizontal position raises the percentage of the blood in it. When in recumbent position the legs are raised passively, the discrepancy between finger and toe percentage becomes still greater, the first rising, the latter falling markedly. But when the erect posture is resumed the normal finger percentage soon returns. Muscular effort always increases the percentage, due to increase in arterial pressure, but is followed by a fall below the normal. Massage and electricity increase the percentage probably by favoring onward flow of lymph and increasing transudation of fluid by augmenting blood pressure. General exercise increases percentage, but reduces worth. Severe prolonged exercise induces profound though temporary changes in worth, but is followed shortly by a general increase. Perspiration and lymph flow have much to do with this. In inactive individuals lowered blood pressure favors storage of tissue fluids, and the blood is poor, while in the regularly active, there is a smaller storage of lymph and the percentage of corpuscles and hemaglobin is permanently high. It seems therefore that in health the composition of the blood varies with the calibre of the arteries, that the variations of the former are largely influenced by the forces of the circulation, and that the physiological relationship between the blood and the circulatory apparatus, is as intimate as is the anatomical.

Considering all this, it becomes very apparent that the mere count of reds and estimation of hemaglobin at arbitrary times without careful consideration of the many factors which normally may influence them, can be little more, perhaps worse than useless.

Of the life history of the white corpuscles, and the occasion of its varied forms, very little is known. Its origin, function and fate are still unsettled, and the changes observed seem to have nothing to do with changes in the red cells. Among the mass of facts recorded about them, there is very little which seems conspicuously useful, principally because different observers have kept things unsettled by giving undue prominence to insignificant peculiarities of morphology and color reactions, and so have been led to give differing interpretations to perhaps the same or at least essentially similar conditions.

To show how important it is to go carefully in forming conclusions from counts, it is only necessary to remember that generally there is a preponderance of a certain type in the adult, while reverse conditions obtain in the young, that there is a constant change in number due to vaso-motor conditions of vessels. That muscular exercise increases them in the puerperal vessels. That during tranquil circulation they idly stick to vessel walls, but are torn loose by increased vigor of the blood current. Cold increases their number in the peripheral vessels. During pregnancy they gradually appear in greater numbers, especially in primipara, and in the new born they are found altogether out of proportion. No one has yet ventured a plausible explanation of these phenomena. In some individuals certain foods, especially albuminoids make the leucocytes appear in greater numbers. Probably this as well as the leucocytosis ordinarily following digestion, may be explained some day by chemotaxis. During digestion they may be observed at different ages to increase from 30 per cent to 100 per cent. and this, like in the case of reds, is probably not an actual change in the total percentage. Certain it is that in a general way, other things being equal, their constant proportion has something to do with the state of nutrition of the individual, and therefore like the red depends on the chemical condition of the plasma and its serum.

It seems plain enough, then, that the effect of different hourly and daily physiological conditions cannot be ignored in estimating the relationship between corpuscles and plasma, chromocytes and leucocytes and perhaps chromocytes and hemaglobin. The ordinary limits of variation must be first established before disease limits that are practically valuable indications can be made.

Now as to the mechanical difficulties. Accuracy in counts can be obtained with the Thoma-Zeiss instrument by being extremely careful in the measurement and dilution of blood drawn into the pipette. Those who have tried it often enough know how careful the operator must be to insure this. Then he must be extremely careful in mounting the drop to be examined. Unless the lines of Newton appear when the cover glass is applied the count will not be true. Moreover a very large number of squares must be counted in two or three drops to get a fair average. Variations of 10 per cent. in the same mixture are not only possible, but common. This implies certainly an hour's work. Add to this the care of the pipette, which is necessary after each operation, to insure good working, and it represents more time than the ordinary practitioner can or does give. In order to get a fair count of leucocytes still greater care in manipulating the larger pipette needed, is required. The bore of this tube is so large that the measurement is more difficult.

Cabott has suggested additional rulings on the counting slide, as shown in this diagram, so that they may be counted at the same time with the reds, and only one sample of blood need be taken. This would simplify matters greatly. The centrifugal method by the hematokrit depends largely in worth on the rapidity with which it can be done. But here there are many sources of trouble and error. In the first place the machine must be carried to the patient and the whirring noise associated with the rapid revolutions must be kept up two minutes. Speed and time must be uniform in every observation to get uniform results. But the greatest trouble arises from the fact that the tube must be entirely filled and placed in the machine before clotting begins. Here, as in the counting, greatest care must be exercised not to squeeze lymph out with the blood, and so attenuate it. Another source of error with the hematokrit, and one that has not been worked up is the varying elasticity and compressibility of the blood cells.

The most useful hemocytometer for ordinary routine is the one devised by Oliver. It is as accurate as any and more easily operated. He explains the principle on which it works after this fashion. When a candle flame is viewed through a glass-tube containing water a transverse line of bright illumination is seen, consisting of closely packed minute images of the flame, produced by the longitudinal fibrillation of the glass. A flat tube works better than a round one. Each corrugation acts as a lense and hence they make together a line of images. When some fixing fluid, like Hayem's is added to the blood it is more or less opaque or laked, according to the proportions of the mixture. This opacity shuts from view the illuminated line until on further diluting a definite point is reached, when it can just be detected as a delicate line across the tube. It was found that the development of this line by dilution is a very delicate indicator of the percentage of red blood corpuscles, and that the white corpuscles if not increased beyond 50 per cent. do not change its accuracy.

A fixed, very small amount of blood is taken in a small capillary tube and the degree of dilution read off from a scale on the mixing tube. This scale is very carefully made with the Thoma-Zeiss instrument. (See cut.) By corking the tube the partially diluted specimen can be carried to the office to be examined at leisure and will keep for twenty-four hours.

Hemaglobin estimation has more of the personal equation in it as a source of error, as well as mechanical. Of course, it can be definitely ascertained by determining the amount of iron in the ash of a fixed volume of blood. But that is not practical, or useful to the clinician.

Spectroscopic estimation is also very accurate, but has the same objection of not being practical. The portable instrument devised by Hönach requires altogether too much blood. This tempts and often necessitates the operator to squeeze the finger in order to get enough, and so a variable amount of lymph is added which cannot be estimated for in the result. The specific gravity method of Hammerschlag is ordinarily quite accurate and requires only a common urinometer outfit. The scale of the float should, however, be accurately standardised. Besides this, only a quantity of benzol and chloroform are needed with an ordinary pipette and stirring rod to complete the outfit. It depends upon the fact that specific gravity of blood runs parallel to the percentage of hemaglobin. Those who have used this method must have found with the writer that here too considerable care is needed to get results. If the inside of the glass is not absolutely dry the blood drops, which it is quite difficult to get into the mixture without air bubbles, will adhere to the side of the tube or pipette. Filling the drops with air can only be avoided by taking quite a quantity of blood with the pipette and so getting sufficient for several drops, one of which may safely be counted on as free of air. The operator must start with a mixture near the specific gravity of blood, else the time required in making the mixture will allow the benzol and chloroform to penetrate the drop, and he will have to begin over. Of course, in conditions in which the plasma is very much changed, *e.g.*, dropsical conditions, pernicious anemia, etc., and even in leukemia, in which the weight of whites interferes, this method is apt to be unreliable. Still, on the whole, it is more generally useful than most colorimetric methods, easier to manipulate and certainly less expensive.

Gower's hemoglobinometer is the simplest and cheapest instrument working on color comparison, but is less accurate than any, because it must be used with daylight. The specific color curve of diluted blood is much more complex with white light than with yellow, such as a candle would give, besides daylight is a varying uncertain thing. In daylight, as you will see from the diagram, the specific color curves of the blood consist of varying proportions of red, orange and yellow. In the lower percentages, 10 to 40, red as a separate color does not exist, it is only present in combination with yellow, as orange, made up of equivalent portions of each. In these lower gradations the place of red is taken by yellow. Between 40 to 50 yellow dies out and then red appears and progressively increases until at the highest grade it is the dominant color. In these daylight gradations the curve of orange is the predominant one, though it begins to die out in the higher part of the scale. In candle light the color curves are quite different and less complicated. Red is predominant throughout, except at the lowest grade, where it is subordinated to orange. From this point upward orange gradually diminishes and vanishes entirely at 90 while pure red distinguishes the highest grades. This remarkable difference is doubtless due to the predominance of yellow emitted from the candle flame.

The Fleischl instrument is the one of this group in most common use, but has the disadvantage of presenting a range of error of from 2 to 20 per cent., apart from the errors which come from differences in color, sensitiveness of different individuals. You are familiar with the instrument. The glass wedge, in the first place, does not correspond through its entire length with the color of corresponding dilutions of blood, especially not if daylight is admitted. Below 20 it is entirely unreliable. Then the portion of glass seen at one time through the cell presents a confusing variation of 20 degrees in its length. This can, however, be obviated by obscuring all but its center.

One of the most distracting features and a great source of error with many in the use of this instrument comes from the fact that blood possesses in a remarkable degree the quality of color, purity or brilliancy, which is a distinct quality from color depth and composition and remains after these have been duly matched, so that the color effect of the dilution can never be made to compare exactly with that of the glass wedge. Add to this necessary source of confusion, a more or less marked degree of lack of color sense, and it is plain that the reading is far too often much of a guess. Almost all these sources of unavoidable error are eliminated in the Oliver hemoglobinometer. Like the Fleischl instrument it requires candle light because of the peculiar color curve of blood. It consists of an automatic blood measure holding 5 c.m.m., which is larger and stronger than that used with the Fleischl. It is also much easier cleaned by drawing a needle and thread through it. A mixing pipette, with rubber nozzle fitting over the measuring tube insuring complete rinsing out of blood with the first few drops of water. A blood cell with cover of lower grade of blue glass, which does away with the confusing brilliancy of the solution and still does not disturb the correct reading. Two sets of standard gradations—six in each—which represents the divisions of 10 degrees of the scale and are carefully standardized. The intermediate points are obtained by riders placed over the standards bringing the reading down to 1 per cent. or less. When they are in use the colorless cover is placed over the cell to balance the effect of the mere layer of glass of the rider. An ordinary small Christmas candle is used for light, excluding all other and the observation is made through a camera tube. It has been found that resting the eye by looking through green glass makes the retina more sensitive to slight differences in color and some instruments have a disk of it attached to the camera tube. The findings with this instrument are most accurate and are quite readily obtained. Unfortunately, it is more expensive than the Fleischl, but much more portable and durable.

It cannot be denied that the examination of blood as it is usually done at the present time, cannot possibly give uniformily reliable results. The clinician must not rely on any and everybody's findings if he would get help and be safe, and especially must he guard against being led astray by the enthusiast.

Nevertheless, any careful, unbiased physician, no matter how busy, who possesses a little knack in handling instruments and the ordinary amount of patience can get very useful information and help by studying the blood in at least all chronic diseases and all disorders associated with fever. A specific gravity outfit for hemoglobin, an Oliver tintometer or hemocytometer, and a box of coverslips, with a microscope, are all he requires to do good work, if he will remember that digestion, exercise, emotional states (vaso-motor disturbances) position, and last but not least, individual peculiarities, etc., have to be considered when interpreting the findings.

I cannot fairly close this subject without saying just a word about the bearing of chemical conditions of the plasma as producing these secondary effects on the cellular elements which we are to some extent able to estimate. We rate the importance of the red cell in the function of exchange of gasses (as far as we know now its only function) altogether too high. It is a fact that reduction of reds and even lessening of lung capacity, if not extreme do not materially modify exchange of gasses. Anemic and leukemic people handle as much O and $CO_2$ as any. Rapid breathing, rapid pulse and lessening of muscle work compensate very efficiently, and yet with this function of the reds within a physiological limit of function, disease processes continue. How then can the mere knowledge of the percentage of cells be of any great value directly, in the treatment of the patient? The health, usefulness and longevity of the red cell, its composition and ability to retain hemaglobin are determined by the chemical condition of the serum. Until we know more about the physiological and pathological chemistry of the blood, we can know little of the meaning of corpuscular changes.

The red cell is very little concerned in carrying $CO_2$. In the normal state it does carry a little on its return voyage, but only an insignificant proportion, some 10 volumes in a 100 of the total excreted. The bulk of $CO_2$ is in loose and firm chemical combination in the carbonates and phosphates of sodium salts in solution in the serum. Even globulin combines to some extent with it. The presence in the serum of the various salts with which it can combine is a much more important matter, and especially their reaction, in order that it may not form firm combination, which the excretory cells of the air vesicles are unable to handle. The presence of phosphoric acid, *e. g.*, is as important here as hemaglobin, whose proteid portion alone only 10 per cent. of its bulk, has anything to do with the elimination of $CO_2$.

In this light how absurd becomes the wholesale administration of iron when hemaglobin, to say nothing of counts, happens to be at one or two arbitrary findings somewhat below the average normal. Yet, 50 per cent. of the blood examinations made today lead to no better scientific result than this in directing or helping the clinician.

A knowledge of the behavior of albumin and globulin, *e.g.*, in wasting diseases, such as chronic infections, malignant tumors or the less serious nutritive changes, and the chemistry of the hydremic conditions, which follow with their effect on the blood cells and their functions would be of infinitely greater help.

To know something of the chemotactic influences of different food stuffs in their absorption and circulating form, as well as that of waste products, which are always in the circulation, and the mark they leave on the corpuscles and their field of operation, would quickly lead us out of the labyrinth and confusion which the sensitive red cell and the mysterious inscrutible noncommunicative white corpuscle in its protean forms and groupings have placed us. It is then to physiolgical chemistry we must look for the key to the situation—the cipher code which will enable us to read clearly the meaning of what we are now able to observe.

519 FRANKLIN STREET.

